# ROR1 Is Expressed in Human Breast Cancer and Associated with Enhanced Tumor-Cell Growth

**DOI:** 10.1371/journal.pone.0031127

**Published:** 2012-03-05

**Authors:** Suping Zhang, Liguang Chen, Bing Cui, Han-Yu Chuang, Jianqiang Yu, Jessica Wang-Rodriguez, Li Tang, George Chen, Grzegorz W. Basak, Thomas J. Kipps

**Affiliations:** Moores UCSD Cancer Center, University of California San Diego, La Jolla, California, United States of America; Sun Yat-sen University Medical School, China

## Abstract

Receptor-tyrosine-kinase-like orphan receptor 1 (ROR1) is expressed during embryogenesis and by certain leukemias, but not by normal adult tissues. Here we show that the neoplastic cells of many human breast cancers express the ROR1 protein and high-level expression of ROR1 in breast adenocarcinoma was associated with aggressive disease. Silencing expression of ROR1 in human breast cancer cell lines found to express this protein impaired their growth *in vitro* and also in immune-deficient mice. We found that ROR1 could interact with casein kinase 1 epsilon (CK1ε) to activate phosphoinositide 3-kinase-mediated AKT phosphorylation and cAMP-response-element-binding protein (CREB), which was associated with enhanced tumor-cell growth. Wnt5a, a ligand of ROR1, could induce ROR1-dependent signaling and enhance cell growth. This study demonstrates that ROR1 is expressed in human breast cancers and has biological and clinical significance, indicating that it may be a potential target for breast cancer therapy.

## Introduction

The receptor-tyrosine-kinase-like orphan receptor 1 (ROR1) was identified by a polymerase chain reaction (PCR)-based search for tyrosine kinases similar to the tropomyocin receptor kinase (Trk) neurotropic receptors [1]. ROR1 and a related protein, ROR2, were identified as orphan receptors with an extracellular Frizzled-like, cysteine-rich domain, an extracellular, membrane-proximal kringle domain, and an intracellular tyrosine-kinase-like domain [2]. Both ROR proteins are evolutionarily conserved among different species [1], [2], [3], [4], [5]. These proteins are primarily expressed during embryogenesis, being most prominent in the developing face, limbs, heart, and lungs. ROR2 knockout mice exhibited dwarfism and cardiac dysfunction leading to neonatal lethality [6], [7]. ROR1-deficient mice, on the other hand, did not exhibit any morphological abnormalities during embryogenesis, but died within 24 hours after birth, presumably due to respiratory failure caused by inadequate development of the muscles required for ventilation [8]. Although mutations in human ROR2 have been implicated in causing certain congenital skeletal defects, including shortened or missing digits and a form of short-limbed dwarfism [9], [10], [11], mutations in ROR1 have not been reported in any human disease.

In prior studies, we and others found that ROR1 was expressed by leukemia cells and some cancer cell lines, and was involved in cell survival [10], [12], [13], [14], [15], [16], [17], [18]. However, it was not known whether other cancers expressed ROR1 or whether its expression had functional and clinical significance. Here, we used a high-affinity mAb specific for ROR1 (named 4A5 [10]) to examine human breast cancers. Our results reveal that human breast cancers express ROR1, which can contribute to tumor-cell growth and survival via activation of PI3K, AKT, and CREB.

## Results

### The Neoplastic Cells of Human Breast Cancers Express ROR1

**Figure 1:**
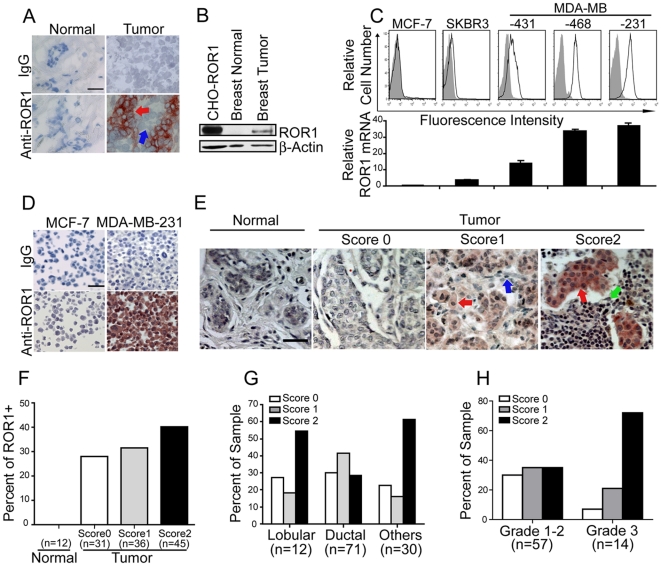
ROR1 is expressed in human breast cancers and breast cancer cell lines. (**A**) Representative images of fresh frozen tissue sections stained for immunohistochemistry with 4A5, a mAb specific for ROR1 (labeled anti-ROR1) or an IgG2b isotype-control mAb (labeled IgG). The bound antibody is red and the nuclear counterstain with hematoxylin is blue. Red arrow points to the tumor cells and blue arrow points to stromal cells. (**B**) Immunoblot analysis of lysates from normal human breast tissue or breast cancer tissue with rabbit antibodies specific for human ROR1 or β-Actin. CHO-ROR1 cells are CHO cells transfected to express human ROR1. (**C**) Fluorescence histograms of human breast cancer cell-lines (MCF-7, SKBR3, MDA-MB-431, MDA-MB-468, or MDA-MB-231) stained with 4A5 (open histograms) or control mAb (shaded histograms) (top panel). The expression of ROR1 mRNA in each tumor cell line relative to MCF-7 cells assessed via quantitative RT-PCR is indicated by the relative height of each bar below the corresponding histogram. The error bars represent the standard error about the mean of triplicate values. (**D**) Formalin-fixed, paraffin-embedded pellets of MCF-7 or MDA-MB-231 cells were stained with 4A5 or control IgG2b. Tissue-bound 4A5 is shown in red and the nuclear counterstain with hematoxylin is in blue (Scale bar in the top left picture represents 35 µm). (**E**) Representative images of breast normal tissues or cancer tissues stained with 4A5 A score of 0 indicates that none of the cells within the sample bound to the anti-ROR1 mAb; a score of 1 indicates low-level binding of the mAb to the tumor cells or low-to-moderate-level binding of the mAb on less than 50% of tumor cells; a score of 2 indicates moderate-level staining on more than 50% of tumor cells or high-level staining of the tumor cells. The scale bar in the top right picture represents 35 µM. Red arrows point to the tumor cells, blue arrow points to stromal cells and green arrow points to lymphocytes. (**F**) The proportion of breast tumor tissues or normal tissues found negative (Score 0) or having weak to moderate staining (Score 1) or strong staining (Score 2) for ROR1 are indicated by in each bar. The number of different cases examined for each tumor type is indicated in the parentheses. (**G**) The proportion of different histological types of breast tumor tissues found lacking staining (Score 0) or having weak to moderate staining (Score 1) or strong staining (Score 2) for ROR1 are indicated by in each bar. The number of different cases examined for each tumor type is indicated in the parentheses. (**H**) The proportion of poorly differentiated (grade 3) or well to moderately differentiated (grade 1–2) ductal breast adenocarcinoma tissues found lacking staining (Score 0) or having weak to moderate staining (Score 1) or strong staining (Score 2) for ROR1 are indicated by in each bar. The number of different cases examined for each tumor type is indicated in the parentheses. Statistical significance of the differences was analyzed using Kruskal-Wallis test (Grade 3 versus grade 1–2, P = 0.013).

Evaluation of fresh-frozen tumor biopsy specimens (N = 4) with 4A5 revealed that breast adenocarcinomas expressed ROR1, in contrast to normal breast tissues (N = 2) which lacks expression of ROR1 [13]. 4A5 also did not bind to stromal cells present in breast tissue (Fig. 1A). A ROR1 protein with a molecular size of ≈120 kD could be detected in tumor-tissue lysates (Fig. 1B). This size was comparable to that of ROR1 identified in Chinese Hamster Ovary (CHO) cells transduced to express human ROR1 (CHO-ROR1) [10]. Many breast cancer cell lines also expressed surface ROR1 (Fig. 1C). On the other hand, some breast cancer cell lines lacked detectable ROR1 (e.g. MCF-7).

4A5 also specifically reacted with ROR1-positive cells that had been fixed in formalin (Fig. 1D). This allowed us to examine tissue microarrays for expression of ROR1 by neoplastic breast cancer specimens of different patients (N = 113) or by normal adult breast tissues (N = 15). The neoplastic cells of high proportions of human breast cancers expressed ROR1, which was not detected on normal breast tissues or the non-neoplastic stromal cells in breast cancer tissue specimens (Fig. 1E–F). The expression levels of ROR1 varied between specimens from different patients. The 4A5 mAb stained the breast cancer cells intensely in forty percent of specimens from different patients (N = 112) and weak to moderately in 33% of the specimens via immunohistochemistry (Fig. 1E–F). We also found that 7 of 12 (55%) patients with lobular breast adenocarcinoma had breast cancer tissue that had high-level expression of ROR1, whereas only 21 of 71 (29%) patients with ductal breast adenocarcinoma had cancer tissue that stained intensely with 4A5 (Fig. 1G). Moreover, 10 out of 14 (72%) patients with poorly differentiated ductal breast adenocarcinoma (grade 3) had cancer tissue that expressed high levels of ROR1 protein. this proportion (72%) was significantly higher than the 19% of patients (N = 57) with moderately-differentiated ductal adenocarcinoma (grade 1–2) that expressed high levels of ROR1 (p = 0.013, Kruskal-Wallis test, Fig. 1H)

### Expression Of ROR1 In Human Breast Cancer Is Associated With Adverse Disease Characteristics

**Figure 2:**
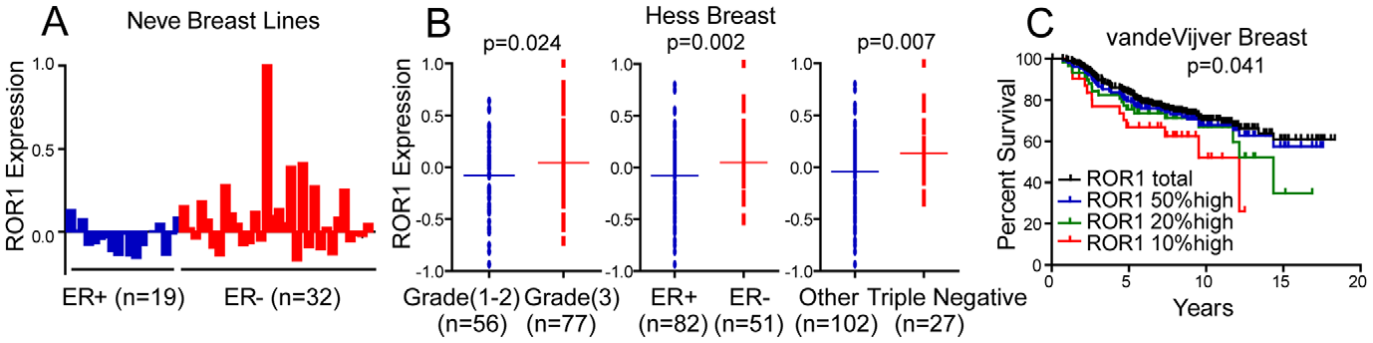
Expression of ROR1 in human breast cancer is associated with aggressive disease. (**A**) Each bar represents median-centered log_2_ expression of ROR1 mRNA by breast cancer cell lines in Vene dataset. Cell lines with similar molecular characteristics are clustered together. (**B**) Each dot plot represents median-centered log_2_ expression of ROR1 mRNA by tumor tissue from an individual patient. Patient samples with similar phenotypic or molecular characteristics are clustered together. The line indicates the median ROR1 expression level by the group. P indicates the statistical significance of the differences in the collective ROR1 expression between the two groups, as calculated using Student's t test. (**C**) Kaplan-Meier survival analysis for 295 breast cancer patients from Vandevijver dataset using defined cut-off values for ROR1 expression (patient sub-groups defined as samples that expressed ROR1 at levels that exceeded that of the mean level of ROR1 expressed by all samples (50% high: n = 148), highest 20% (20% high: n = 58) or highest 10% (10% high: n = 30) relative ROR1 expression. Statistical difference was determined by log-rank test. The each dataset used is indicated on the top of each graph.

We found ROR1 was expressed on the estrogen-receptor-negative breast cancer cell lines MDA-MB-231 or MDA-MB-468, but not on the estrogen-receptor-positive breast cancer cell lines MCF-7 or SKBR3 [19] (Fig. 1C). Because of this, we interrogated published DNA microarray datasets on primary human breast cancers and cancer cell lines for expression of ROR1 and hormone receptors [19], [20], [21]. We noted that many of the breast cancer cell lines included in a dataset published by Neve and colleagues [19] had levels of *ROR1* comparable to that of MDA-MB-231 or MDA-MB-468. These ROR1-positive cell lines generally also lacked expression of estrogen receptors, or estrogen/progesterone receptors and HER2/Neu (triple-negative) (Fig. 2A and Fig. S1A). We also found that primary breast cancer tissues that either were poorly differentiated (grade 3), lacked expression of estrogen receptors, or were triple negative, generally expressed higher levels of *ROR1* than primary breast cancer tissues without such poor-prognostic features (Fig. 2B, S1B–C). Finally, Kaplan-Meier survival analysis revealed an association between higher expression levels or ROR1 and shorter overall survival times (p<0.05) (Fig. 2C). These data suggest that breast-cancer expression of *ROR1* is a characteristic associated with poorly differentiated breast cancers that generally have an adverse clinical outcome.

### ROR1 Promotes Tumor-Cell Survival And Growth

**Figure 3:**
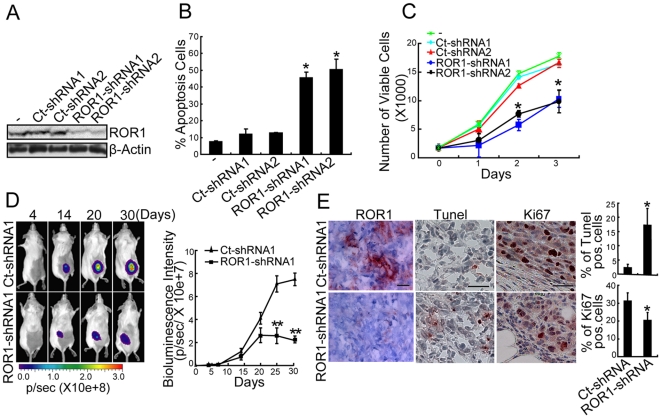
Expression of ROR1 promotes tumor-cell survival and growth. (**A**–**B**) MDA-MB-231 cells transiently transduced with control shRNA (Ct-shRNA1 or 2) or ROR1-shRNA1 or 2, as indicated at the top of each lane, were examined for ROR1 expression by immunoblot analysis (A) or stained with Annexin V for apoptosis detection after 2 days (B). The height of each bar in the graph B indicates the mean proportion of cells that were Annexin-V positive. The error bars indicate the standard error of triplicate samples. P indicates the statistical significance as measured by Dunnett's multiple comparisons test. _*_ indicates P<0.05. (**C**) MDA-MB-231 cells were transduced with vectors encoding Ct-shRNA or ROR1-shRNA and then selected for stable expression of the shRNA. Equal numbers of cells indicated in the legend provided in the top left corner were cultured and monitored for growth over time. Each graph indicates the average number of viable cells assessed at 0, 1, 2, or 3 days using the WST-8 assay. The error bars indicate the S.E. of the mean for triplicate samples. _*_ indicates P<0.05 and _**_ indicates P<0.01(Student's t test). (**D**) Representative bioluminescent imaging of RAG^−/−^/γ_c_^−/−^ mice engrafted with luciferase-labeled MDA-MB-231 cells transduced with Ct-shRNA1 (top row) or ROR1-shRNA1 (bottom row). The intensity of the fluorescence signal related to cell number is indicated by the scale on the bottom. The graph on the right provides the mean fluorescence activity of RAG^−/−^/γ_c_^−/−^ mice engrafted with luciferase-labeled MDA-MB-231 cells transduced with Ct-shRNA1 (closed triangles) or ROR1-shRNA1 (open triangles) at various times after engraftment, as indicated by the legend in the upper left corner. The error bars indicate S.E.M. fluorescence activity of groups of mice (n = 5–7) at each time point. _**_ indicates P<0.01(Student's t test). (**E**) Tumors were extracted from mice engrafted with MDA-MB-231 cells transduced with Ct-shRNA1 or ROR1-shRNA1 and examined for ROR1 (left), TUNNEL (middle), or Ki-67 (right). The scale bar in the top right panel corresponds to 35 µm. The nuclear counterstain is in blue and antibody staining is in red. The height of each bar on the right graph indicates the proportion of Tunel-positive or Ki67-positive cells in tumors that formed from MDA-MB-231 cells transduced with either Ct-shRNA or ROR1-shRNA. Error bars indicate S.E.M (n = 7). * indicates P<0.05 (Student's t test).

We transduced MDA-MB-231 with vectors encoding short-hairpin RNAs that could either specifically silence ROR1 (ROR1-shRNA) or serve as a control (Ct-shRNA). We found that MDA-MB-231 cells had higher proportions of cells undergoing apoptosis following silencing of ROR1 than MDA-MB-231 cells transduced with Ct-shRNA (Fig. 3A–B). We selected cells of MDA-MB-231 silenced for ROR1 that maintained negligible expression of ROR1 (Fig. S2A). MDA-MB-231 cells silenced for ROR1 had a significantly smaller BrdU-labeling index than did cells cultured from MDA-MB-231 cells that were transduced with Ct-shRNA and maintained expression of ROR1 (Fig. S2B). MDA-MB-231 silenced for ROR1 also had reduced growth rates relative to that of MDA-MB-231 cells treated with Ct-shRNA (Fig. 3C).

Control-treated and ROR1-silenced MDA-MB-231 cells were transfected with a lentivirus encoding GFP and luciferase and selected for expression of GFP [22]. When equal numbers of such cells were implanted into female immune deficient RAG^−/−^/γ_c_^−/−^ mice we observed significant differences in their mean fluorescence intensities at three weeks. Moreover, the mean fluorescence intensity of the tumors derived from control-treated cells was 4 times greater than that of tumors silenced for ROR1 when imaged thirty days after implantation (Fig. 3D). Tumors from cells silenced for ROR1 had lower ROR1 expression, 5 times more apoptotic cells, and significantly lower proportions of cells that stained for the proliferation marker Ki-67 than did tumors derived from control-treated tumor cells (Fig. 3E).

### Expression Of ROR1 Enhances Activation Of CREB

**Figure 4:**
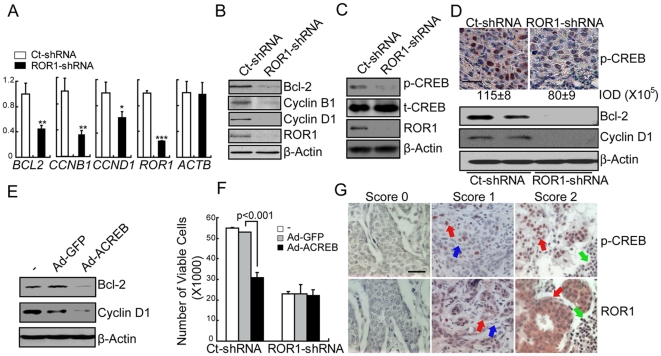
ROR1 activates CREB to regulate tumor-cell growth. (**A**) Relative expression of selected genes by MDA-MB-231 cells transduced with either Ct-shRNA (white bars) or ROR1-shRNA (black bars), as assessed via quantitative RT-PCR. The numbers on the y-axis represent fold difference in specific gene expression relative to GAPDH. Error bars indicate S.E.M. (n = 3 experiments). _*_ indicates P<0.05, _**_ indicates P<0.01 and _***_ indicates P<0.001 (Student's t test). (**B**) Immunoblot analyses for proteins listed on the right margin, using lysates from MDA-MB-231 control cells versus cells silenced for ROR1. (**C**) Immunoblot analysis of CREB phosphorylation at ser-133 (p-CREB), total CREB (t-CREB), ROR1, or β-Actin for MDA-MB-231 cells with or without ROR1 silencing, as indicated on the top of each panel. The phospho-specific antibody recognizes phospho-serine-133 CREB. (**D**) Tumors extracted from mice engrafted with MDA-MB-231 control cells or ROR1-shRNA-transduced cells were examined for p-CREB by immunohistochemistry staining (n = 3) or Bcl-2, Cyclin D1 and β-Actin protein expression by immunoblot analysis. The scale bar in the left panels represents 35 µm. The antibody staining is in red and the nuclear counterstain is in blue. The intensity of p-CREB was quantified and data were represented as mean intensity ± S.E.M from 3 tumor tissues per group. (**E**) MDA-MB-231 cells infected with Ad-GFP or Ad-ACREB were examined for expression of proteins by immunoblot analyses. (**F**) MDA-MB-231tumor cells with or without ROR1 expression were transfected with Ad-GFP or Ad-ACREB and then monitored for cell growth. The numbers of viable cells (after 72 hours culture) are represented by the height of each bar in the graph. The error bars provide the S.E.M. of triplicate samples. P indicates the statistical significance as assessed by Student's t test. (**G**) Representative images of breast cancer tissues stained for phospho-CREB (p-CREB) (top panels) or ROR1 (bottom panels). Tissue-bound antibody is shown in red and the nuclear counterstain with hematoxylin is in blue. The scale bar in the top left picture represents 35 µM. Tissues were scored as 0 (none of the cells within the sample bound to the mAb); 1 (low-level binding of the mAb to the tumor cells or low-to-moderate-level binding of the mAb on less than 50% of tumor cells); 2 (moderate-level staining on more than 50% of tumor cells or high-level staining of the tumor cells). Red arrows point to the tumor cells, blue arrow points to stromal cells and green arrow points to lymphocytes.

We examined the gene expression profiles of MDA-MB-231 cells silenced for ROR1 or transduced with Ct-shRNA. We adopted a network-based classification scheme that combines gene expression measurements over groups of genes that fall within common pathways [23]. Cells silenced for ROR1 had lower expression levels of genes encoding proteins induced by CREB than control-treated cells (Fig. S3A). The transcriptional expression levels of 38% of all CREB bound genes were decreased in cells silenced for ROR1 relative to that of control cells. Cells silenced for ROR1 had reduced expression of 45% or 40% of the CREB-bound genes encoding proteins involved in cell proliferation or apoptosis, respectively (Fig. S3B–C and Table S1). Moreover, MDA-MB-231 cells silenced for ROR1 had relatively low expression of 70% of the CREB-bound genes in 29 of 49 genes contained with the 70 gene prognostic-signature associated with tumor specimens from patients with aggressive disease [24] (Fig. S3D and Table S2). Quantitative RT-PCR analysis validated these findings for selected genes. In particular, we found that cells silenced for ROR1 had lower expression levels of *CCNB1*, *BCL2*, and *CCND1* than did control-treated cells (Fig. 4A). The proteins encoded by these genes also were expressed at lower levels in MDA-MB-231 cells silenced for ROR1 than in control-treated cells (Fig. 4B).

We examined cells for phosphorylated CREB (p-CREB) at serine-133 (Ser-133), which is a feature of activated CREB [25]. MDA-MB-231 cells silenced for ROR1 had significantly lower levels of p-CREB relative to that of MDA-MB-231 cells that were transfected with Ct-shRNA (Fig. 4C). Lower levels of p-CREB also were found in tumors that developed from cells silenced for ROR1 than in tumors that developed from control-treated cells (Fig. 4D, upper panel). Furthermore, expression of CREB-target genes was reduced in tumors derived from MDA-MB-231 cancer cells than that in tumors silenced for ROR1 (Fig. 4D, lower panel).

We also transduced MDA-MB-231 cells with an adenovirus vector encoding either a dominant-negative CREB (Ad-ACREB) or green fluorescence protein (Ad-GFP). Cells transduced with Ad-ACREB had reduced expression of CREB-target genes than cells transduced with Ad-GFP (Fig. 4E). Moreover, after three days, cultures of tumor cells transduced with Ad-ACREB had significantly lower proportions of viable cells than cultures of cells transduced with Ad-GFP. On the other hand, there were no apparent differences in growth of cells silenced for ROR1 following transduction with Ad-ACREB or Ad-GFP (Fig. 4F). Similar effects were obtained when CREB was silenced using CREB siRNA (Fig. S4A). Finally, we examined paraffin-embedded primary human breast tumor tissues for ROR1 and p-CREB, and scored them on the basis of their intensity of staining for ROR1 or p-CREB. We observed a significant association between expression of ROR1 and p-CREB in primary tumors (N = 36, P<0.05, χ^2^ test, Fig. 4G and Table S3). Collectively, these results indicate that activation of CREB is associated with expression of ROR1 in MDA-MB-231.

**Figure 5:**
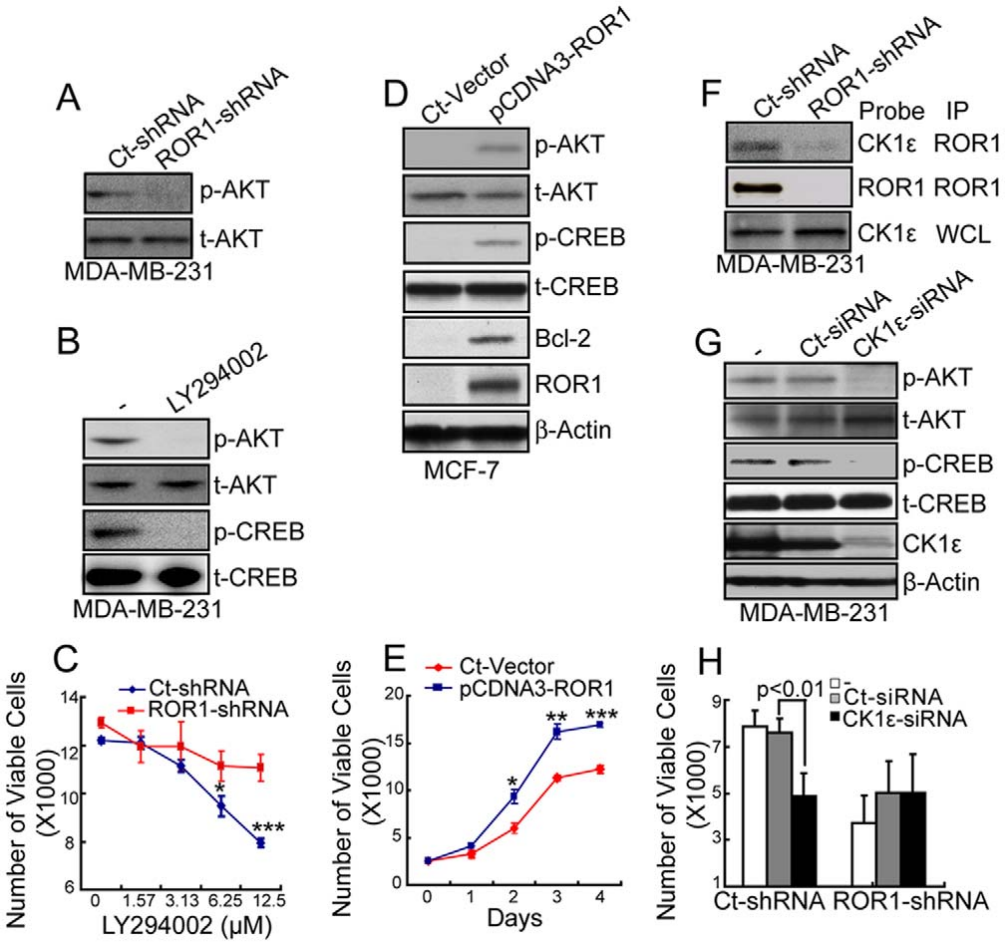
ROR1 interacts with CK1ε to activate PI3K/AKT, leading to activation of CREB. (**A**) MDA-MB-231tumor cells with or without ROR1 expression were examined for phosphorylated AKT at ser-473 (p-AKT) or total AKT (t-AKT). (**B**) MDA-MB-231 cells were cultured with (+) or without (−) LY294002 and examined for p-AKT, t-AKT, p-CREB, or t-CREB by immunobot analysis after 16 hours. (**C**) MDA-MB-231 cells (Ct-shRNA) or cells silenced for ROR1 (ROR1-shRNA) were cultured with different concentrations of LY294002 and monitored for cell growth after 48 hours using the WST-8 assay. The graphs depict the mean numbers of viable cells, ± S.E.M of triplicate samples, which are representative of three independent experiments. *P<0.05 and ***P<0.001). (**D–E**) MCF7 control cells (Ct-Vector) or cells made to express ROR1 (pCDNA3-ROR1) were examined for protein expression (D) or (E) monitored for growth over 4 days using the WST-8 assay. The graph depicts the mean numbers of viable cells ± S.E.M. over time in each of these cell lines, as indicated in the legend at the top of the figure, which are representative of three independent experiments. *P<0.05, **P<0.01 and ***P<0.001 by Student's t test. (**F**) Protein lysates from MDA-MB-231 cells or cells silenced for ROR1 were immunoprecipitated (IP) with ROR1 antibody. The bound immunoprecipitated products and whole cell lysate (WCL) were probed by the antibodies indicated in the Probe column. (**G–H**) MDA-MB-231 cells were treated without (−) or with CK1ε siRNA (CK1ε-siRNA) or control siRNA (Ct-siRNA) for 72 hours and examined for protein expression (G) or cell growth (H). The height of each bar in the graph F provides the mean number of viable cells ± S.E.M., which are representative of more than three independent experiments. P indicates the statistical significance.

We examined for activation of signaling proteins upstream of p-CREB, such as AKT or phosphoinositol 3′ kinase (PI3K) [26]. We found MDA-MB-231 cells silenced for ROR1 had lower levels of p-AKT relative to AKT than control-treated cells (Fig. 5A). On the other hand, treatment with a PI3K inhibitor (LY294002) reduced the levels of p-AKT and p-CREB in MDA-MB-231 cells that expressed ROR1 (Fig. 5B). Moreover, ROR1-expressing MDA-MB-231 cells were more sensitive to the growth-inhibitory activity of LY294002 than MDA-MB-231 cells silenced for ROR1 (Fig. 5C).

A similar relationship between p-CREB and ROR1 was observed in MCF-7 cells transduced to express ROR1. MCF-7 made to express ROR1 had increased levels of p-AKT and p-CREB, greater expression of CREB-target genes, and more rapid cell growth in vitro than ROR1-negative MCF-7 cells transduced with a control vector (Fig. 5D–E).

We immune-precipitated ROR1 in lysates of MDA-MB-231 cells and evaluated whether the ROR1-immune precipitate contained casein kinase 1 epsilon (CK1ε), a kinase found capable of associating with ROR2 [27] that may activate AKT [28]. We found ROR1 was associated with CK1ε (Fig. 5F). Inhibiting expression or function of CK1ε by respective treatment of MDA-MB-231 cells with CK1ε siRNA or a CK1ε inhibitor, IC261, reduced the levels of p-AKT and p-CREB relative to AKT and CREB (Fig. 5G, Fig. S4B). Such treatment also significantly reduced the growth of MDA-MB-231 cells, but had less effect on MDA-MB-231 cells previously silenced for ROR1 (Figure 5H, Fig. S4C).

### Role Of Wnt5a In ROR1-Dependent Activation Of PI3K/AKT/CREB Signaling And Enhanced Tumor Growth

**Figure 6:**
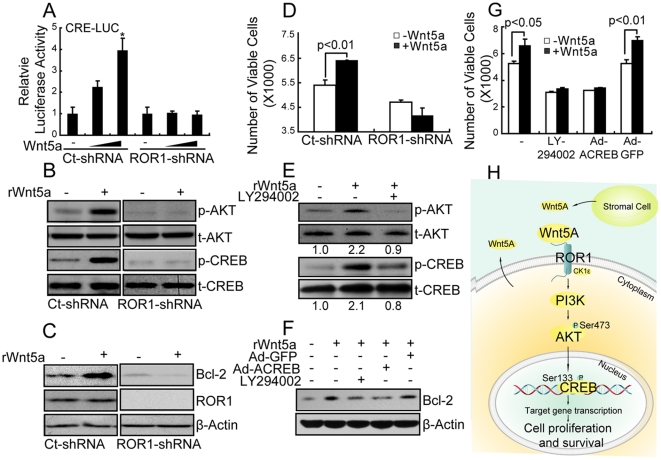
Wnt5a is involved in ROR1-mediated signaling and cell growth. (**A**) MDA-MB-231 cells (Ct-shRNA) or cells transduced for ROR1-shRNA (ROR1-shRNA) were transfected with Cre-luciferase reporter construct and/or increasing amounts of Wnt5a expression vector. The bars provide the mean relative luciferase activity of the various cell populations. *P<0.05 by Dunnett's multiple comparisons test. (**B–D**) MDA-MB-231 cells or cells silenced for ROR1 were stimulated with/without 250 ng/ml exogenous recombinant Wnt5a protein (rWnt5a) and examined for p-AKT, AKT, p-CREB, CREB at 15 minutes (B), BCL-2, ROR1 and β-actin expression at 48 hours (C) and cell growth at 48 hours (D). The graph depicts the mean numbers of viable cells ± S.E.M., which is representative of more than three independent experiments. (**E**) MDA-MB-231 cells were pretreated with/without 10 µM LY294002 for 2 hours and then stimulated with/without 250 ng/ml rWnt5a for 15 minutes and examined for protein expression. Relative phospho-protein levels were normalized to total-protein. (**F–G**) MDA-MB-231 cells were treated with/without 10 µM LY294002, or infected with either Ad-GFP or Ad-ACREB, or cultured in the media with/without 250 ng/ml rWnt5a for 48 hours as indicated on the top, and examined for proteins (F) or cell growth using WST-8 assay (G). The height of each bar in the graph provides the mean number of viable cells ± S.E.M. in each cell population. P indicates the statistical significance as determined using Student's t test. (**H**) Model of ROR1 promoting tumor-cell growth. Wnt5a expressed by tumor or tumor microenviroment (e.g. stromal cell) interacts with ROR1 to activate PI3K/AKT through CK1ε, leading to activation of CREB to enhance expression of genes that enhance resistance to tumor cell apoptosis and/or promote tumor cell growth.

Prior studies demonstrated that ROR1 could serve as a receptor for Wnt5a [10]. To evaluate the response of ROR1 to this ligand in breast cancer cells, MDA-MB-231 cells that express low-levels of Wnt5a (Fig. S5A) were transfected with increasing amounts of vector encoding human Wnt5a together with a vector encoding a Cre-luciferase reporter construct. The relative luciferase activity was increased by transfection of MDA-MB-231 cells with the Wnt5a construct in a dose dependent fashion (Fig. 6A). This effect was not observed using MDA-MB-231 cells previously silenced for ROR1 (Fig. 6A). Treatment of MDA-MB-231 cells with recombinant Wnt5a (rWnt5a) also enhanced the levels of p-AKT and p-CREB, and induced higher expression of CREB-target genes in MDA-MB-231 cells, but not in cells silenced for ROR1 (Fig. 6B–C). Furthermore, treatment of cells with rWnt5a increased the number of viable MDA-MB-231 cells in a dose-dependent manner (Fig. S5B), but not MDA-MB-231 cells lacking ROR1 (Fig. 6D). Treatment of MDA-MB-231 cells with LY294002 could block Wnt5a-induced phosphorylation of AKT and CREB (Fig. 6E). The capacity of Wnt5a to enhance expression of CREB target genes or to promote tumor-cell growth also was inhibited by treatment with LY294002 or transduction of the cells with Ad-ACREB (Fig. 6F–G).

## Discussion

Here we present data demonstrating that high proportions of human breast cancers expressed ROR1. The expression level of ROR1 varied among patient samples; whereas some cases had uniform high-level expression of ROR1, others cases had low or non-detectable levels of this protein. In any case, a sizable fraction of breast cancers expressed ROR1, which was in contrast to the non-neoplastic normal breast tissues. Consistent with these findings, we found that many established breast cancer cell lines express ROR1. This was corroborated by examination of the gene expression data on breast cancer cell lines published by Neve et al [19], [29], [30], [31]]. Moreover, the breast cancer cells that expressed ROR1 generally lacked expression of hormone receptors and/or HER2/neu, which was in contrast to most breast cancer cells lines found to lack expression of ROR1.

Two individual gene expression data were used to evaluate whether breast cancers that expressed *ROR1* had clinical significance distinct from that of ROR1-negative tumors. The patients from Vandevijver dataset had stage I or II breast cancer and were younger than 53 years of age. The predictive power of the prognosis profile was evaluated using either univariable or multivariable statistical analyses. On the other hand, the patients from the Hess breast cancer study had stage I–III breast cancer [20], [21]. In either dataset, we found that primary breast cancers that expressed high-levels of ROR1 were more likely to lack expression of estrogen or progesterone receptors or HER2/Neu, consistent with the observation in breast cancer cell lines. Conceivably, tumors that are poorly differentiated are more likely to express ROR1, a protein that normally is expressed by embryonic cells. Patients who had primary breast cancers that expressed higher levels of ROR1 had a significantly shorter median survival than did patients with primary breast cancers that had low-to-negligible expression of ROR1. As such, expression of ROR1 appears associated with aggressive disease in human breast cancer.

We silenced ROR1 expression in breast cancer cell lines to evaluate its function on tumors. When silenced for ROR1, cancer cells were more sensitive to spontaneous apoptosis and had an impaired cell growth. On the other hand, MCF-7 cells that were transduced to express ROR1 had more aggressive growth characteristics than did control MCF-7 cells. Collectively, our studies indicate that expression of ROR1 is conducive to cancer-cell growth.

One study found that the activity of ROR1 in NCI-1993 cells was dependent upon the expression of *MET*, a proto-oncogene that encodes the hepatocyte growth factor receptor (HGFR) [18]. However, the MDA-MB-231 or MCF-7 cells used in the current study respectively expressed low or negligible levels of *MET* (Fig. S6), making it less unlikely that expression of *MET* is required for ROR1 function. Moreover, the neoplastic cells in chronic lymphocytic leukemia also appear dependent upon expression of ROR1 [17], but do not express *MET* (Fig. S6), suggesting that the function ROR1 also does not require HGFR in primary tumor cells.

Instead, the current study highlights an important role played by CREB, a transcription factor that is over-expressed and constitutively phosphorylated in a number of human cancers and that appears to play a direct role in tumor pathogenesis and prognosis [32], [33], [34], [35], [36], [37], [38]. MDA-MB-231 cells silenced for ROR1 expressed lower levels of CREB-target genes, many of which are expressed by breast cancers of patients with aggressive disease [21], [24]. Furthermore, transduction of cancer cells with the dominant negative CREB (ACREB) inhibited expression of CREB-target genes and reduced the growth of cancer cell lines that expressed ROR1, implying that activation of CREB contributes to the enhanced tumor-growth characteristics afforded by expression of ROR1.

Prior studies found that activation of AKT could directly induce phosphorylation of CREB at Ser-133 [26]. We found that AKT was activated in cancer cells that expressed ROR1, but not in cells that were silenced for ROR1. We also found ROR1 could associate with CK1ε, which was reported to promote the activation of AKT and the survival and proliferation of cancer cells [28], [39]. Treatment of MDA-MB-231 cells with inhibitors of PI3K or CK1ε, or silencing expression of CK1ε, also could impair activation of CREB and reduce the numbers of viable cells relative to that of control-treated cells. Blocking PI3K/AKT signaling also blocked the capacity of Wnt5a to enhance phosphorylation of AKT or CREB, expression of CREB target genes, or cell growth.

Previously we found that Wnt5a could bind ROR1 and enhance the survival of CLL cells, which expressed ROR1, but lacked expression of Wnt5a [40]. Because marrow stromal cells capable of supporting CLL cell survival express Wnt5a, we hypothesized that Wnt5a could function in a paracrine manner to support CLL-cell survival. Indeed, in this study, we found that exogenous Wnt5a could enhance the phosphorylation of AKT and CREB, the expression of CREB-target genes, and the growth of tumor cells that expressed low-levels of Wnt5a, indicating that Wnt5a could act in a paracrine manner for such tumors [41]. However, because some cancer cells have been noted to express Wnt5a [42], [43], it is conceivable Wnt5a also might serve as an autocrine that can enhance the growth of turmors that co-express ROR1.

Based upon these collective results we propose that ROR1 interacts with CK1ε to activate PI3K/AKT/CREB, which in turn induces expression of genes that can enhance resistance to apoptosis and/or promote tumor cell growth (Fig. 6E). The results presented here indicate that a substantial subset of human breast cancers expresses ROR1 and such expression may be associated with aggressive disease. Because of the selective expression of ROR1 by neoplastic cells and its apparent role in promoting tumor-cell growth, ROR1 may serve as a potential target for development of anti-cancer therapies.

## Methods

### Ethics statement

The project was reviewed and approved by one of UCSD's Institutional Review Board (IRB) in accordance with the requirements of the Code of Federal Regulations on the Protection of Human subjects (45 CFR 46 and 21 CFR 50 and 56), including its relevant subparts. **IRB#080918**

All experiments on mice were conducted in accordance with the guidelines of National Institutes of Health (NIH; Bethesda, MD, USA) for the care and use of laboratory animals. The study protocol was approved by UCSD and Medical Experimental Animal Care Committee (USA). **Approval#: S02145**

### Cell culture

Cancer cell-lines were purchased from the American Type Culture Collection (ATCC, Manassas, VA, USA). All cell-lines were cultured at 37°C in a 5% CO_2_/95% humidified air incubator in Dulbecco's modified Eagle medium (DMEM, Invitrogen, Carlsbad, CA) with 10% fetal bovine serum (FBS, Invitrogen), 50 µg of penicillin G, and 50 µg of streptomycin sulfate. We added puromycin (1 µg/ml) to MDA-MB-231 cells or G418 (1.5 µg/ml) to MCF-7 cells transduced with lentivirus encoding Ct-shRNA or ROR1-shRNA, or transfected with pCDNA3-ROR1 or pCDNA3. Stable transfectants were selected by flow cytometry.

### Immunohistochemistry

Tissue microarray (TMA) slides and frozen tissues were purchased from the National Development and Research Institutes, Inc. (NDRI, New York, NY) or Cooperative Human Tissue Network (CHTN, Virginia) Tissue Microarray.. After IRB approval, immunohistochemical staining (IHC), using DAKO Universal LSAB2 kit (DAKO, Denmark), was performed to detect ROR1, Ki67, or phosphor-CREB. Tunel staining was performed with the ABC Vecrastain kit (Vector) and the AEC-cobalt-nickel staining kit according to the manufacturer's directions. The level of ROR1 or p-CREB was scored on the following scale: A score of 0 indicates that none of the cells within the sample bound to the anti-ROR1 mAb; a score of 1 indicates low-level binding of the mAb to the tumor cells or low-to-moderate-level binding of the mAb on less than 50% of tumor cells; a score of 2 indicates moderate-level staining on more than 50% of tumor cells or high-level staining of the tumor cells. Scoring of the tissue microarray was performed in a blinded-fashion by a board-certified pathologist. The signal intensity of phosphor-CREB for tumors xenografted in mice was determined by Image-Pro Plus image analysis software following a previous method [44]. The integrated optical density (IOD) represented averages from 10 non-overlapping images of each tumor specimen. All quantitative studies were performed blinded with regards to animal genotype.

### Cell proliferation assay

Cell proliferation was analyzed using “Cell-Counting Kit Solution” (CCK-8) (Dojindo, Kumamoto, Japan). Cells were plated in 96-well plates at 1–5×10^3^ cells/well and maintained at 37°C in a humidified incubator with vehicle or recombinant Wnt5a protein (R & D Systems, Minneapolis, MN). 10 µl of CCK-8 was added to each well at different time points and cells were incubated for 3 hours. The absorbance at 450 nm was measured to calculate the numbers of viable cells in each well.

### Silencing of human ROR1, CK1ε, or CREB

Virapower lentivirus expression system (Invitrogen) was used for the expression of shRNA according to the manufacturer's instructions. The constructs of ROR1-shRNA1 and Ct-shRNA1 encode red fluorescence protein (RFP). Oligonucleotides for ROR1-shRNA1 and non-targeting (Ct-shRNA1) construct were synthesized (Integrated DNA Technologies) and inserted into the RFP-pLKO.1 vector. ROR1-shRNA2 and non-targeting (Ct-shRNA2) constructs were purchased from Open Biosystems (Rockford, IL). CK1ε siRNA or non-targeting (control) were synthesized by Invitrogen, CREB1 siRNA were purchased from Ambion. All siRNA transfections were performed in DMEM serum-free medium using lipofectaimine RNAiMAX (Invitrogen) according to the manufacturer's instruction.

### Flow cytometry

4A5 or a control mouse IgG2b was conjugated with Alexa Fluor 647 as described [10]. Cells were incubated with antibodies for 20 min at 4°C in staining media (PBS containing 3% FBS and 1 mM 4-(2-hydroxyethyl)-1-piperazine-ethanesulfonic acid (HEPES), pH = 7.4). After that cells were washed twice prior to analysis on a FACS-Calibur (Becton-Dickinson, San Jose, CA) using FlowJo software (Tree Star, Inc., Ashland, OR). To evaluate for apoptosis, cells were stained with 5 µg/ml Annexin V-FITC (Biovision, Mountain View, CA) in the dark for 15 min at room temperature.

### Immunoblot

Human tissues, CLL cells isolated from patients or cell lines were lysed in buffer containing 1% NP40, 0.1% SDS, 0.5% sodium deoxylate, and protease inhibitors in phosphate buffered saline (PBS, pH = 7.2). Size-separated proteins were transferred to membranes, which then were incubated with primary antibodies specific for ROR1, Cyclin D1, Bcl-2, Cyclin B1, phosphor-CREB (ser-133), CREB, phosphor-AKT (Ser-473), AKT (Cell Signaling Technology, Danvers, MA), Met (R & D Systems, Minneapolis, MN), Casein kinase 1 epsilon, Wnt5a, or β-Actin (Santa Cruz Biotechnology, Santa Cruz, CA). After washing the membranes, they were incubated with secondary antibodies that were conjugated with horseradish peroxidase. Blots were then prepared for enhanced chemiluminescence and subsequent autoradiography. The protein concentration was determined using a bicinchoninic acid protein assay (Pierce, Rockford, IL).

### Quantitative RT-PCR

Total RNA was prepared from cells using RNeasy Mini kit (Qiagen, Valencia, CA). One µg of RNA was reverse transcribed at 42°C for 45 min in a 20 µl of reaction mixture using the Reverse Transcription System (Promega, Madison, WI). The reaction mixture for PCR contained 0.2 µm of each primer (see supplemental experiment procedure), 30 ng of cDNA and 10 µl of SYBR Green qPCR Kit (Applied Biosystems). Real-time quantitative PCR was performed for 40 cycles of 15 seconds at 95°C and 60 seconds at 60°C, using an ABI 7700 sequence detection system (Applied Biosystems). The amplified product was determined by comparing the Ct values of each sample to a standard curve and normalized by a housekeeping gene, GAPDH. Values are expressed as fold increase relative to that observed in Ct-shRNA MDA-MB-231 cells.

### Luciferase assay

MDA-MB-231 cells were seeded in 24 well plates. After 24 hours culture, the cells at 70% confluence were transfected with 0.2 µg Cre-luciferase reporter, 0.05 µg control plasmid pCMX-β-galactosidase, and 0.1–0.2 µg pCDNA3.1-Wnt5a in lipofectamine 2000 (Invitrogen). Forty-eight hours after transfection, cells were harvested for determination of luciferase activity using a luminometer (MicroBeta TriLux, Gaithersburg, MD). The luciferase values were normalized for variations in transfection efficiency by using beta-galactosidase as an internal control, expressed as relative luciferase activity, and compared with the designated control cultures. All assays were performed in triplicate.

### Immune-deficient mice and growth of human tumor xenografts

Six to eight week old BALB/c RAG^−/−^/γ_c_^−/−^ mice were obtained from Dr. Catriona Jamieson (University of California, San Diego) and housed in laminar-flow cabinets under specific pathogen-free conditions and fed *adlibrium*. All experiments on mice were conducted in accordance with the guidelines of National Institutes of Health (NIH; Bethesda, MD, USA) for the care and use of laboratory animals. The study protocol was approved by UCSD and Medical Experimental Animal Care Committee (USA). Subconfluent MDA-MB-231 cells transduced with Ct-shRNA1 or ROR1-shRNA1, with lentivirus containing GFP and luciferase, were treated with trypsinized, suspended in serum-free medium (5×10^6^ cells/ml), and mixed with an equal volume of cold Matrigel. Each cell suspension (10^6^ cells in 400 µl) was injected into the subcutaneous tissue of female RAG^−/−^/γ_c_^−/−^ immune-deficient mice. We detected luciferase signaling in mice implanted with cells that were transduced to express luciferase using a sensitive in vivo imaging system (IVIS 200 series, Xenogen, Alameda, CA), described by Rice and colleagues [45]. Mice were anesthetized with isoflurane and injected with luciferin (150 mg/kg intraperitoneal (i.p.) injection) approximately 10 min before imaging. The total photon flux emission (photons/s) from the region of interest (ROI) covering the entire tumor was analyzed with Living Image™ software (Caliper Life Science, Hopkinton, MA).

### Statistical analyses

The standard χ^2^ test was used to analyze for correlation between p-CREB expression and ROR1 expression in human breast tissues. ROR1 expression levels in breast cancer patients sample with different phenotypic or molecular character were analyzed by a two-tailed Student's t test, using either the log ratios of gene expression values without modification [21] or the transformed log ratio of the average intensity of ROR1 expression values [20]. The survival curves of the resulting four groups were compared using the log rank test. A two-tailed Student's t test also was used to examine for statistical differences in levels of apoptosis, proliferation, or rates of engraftment in RAG^−/−^/γ_c_^−/−^ immunodeficient mice, of paired sample populations of cells transduced with Ct-shRNA versus ROR1-shRNA. A one-way analysis of variance (ANOVA) followed by Dunnett's multiple comparisons test was used to evaluate the statistical difference in rates of proliferation or in the luciferase activity for cells. All analyses were performed using Graphpad Prism software (GraphPad Software, La Jolla, CA). A P value of less than 0.05 was considered significant.

### BrdU incorporation assay

The level of DNA synthesis was determined by measuring the amount of 5-bromo-2′-deoxyuridine (BrdU) incorporated during a set labeling period. The cells were starved through culture in serum-free medium for 36 hours, isolated, and then reseeded in 6-well plates for culture overnight in culture media. Then 10 µM BrdU was added to each well and the cells were incubated for 2 hours. After several washes with PBS, the cells were fixed with 4% paraformaldehyde for 30 minutes and then stained with anti-BrdU-FITC antibody and 7-AAD for cytometry, using procedures recommended by the manufacturer (BrdU flow kit, Becton Dickinson, Mountain View, CA).

### Expression Microarray Analysis

Total mRNA from Ct-shRNA MDA-MB-231 stable cell line and ROR1-shRNA MDA-MB-231 stable cell line were labeled with Cy5, hybridized to a human Oligo Microarray (Phalanx Human Whole Genome OneArrayTM, Phalanx Biotech) according to the manufacturer's protocol. Arrays in duplicates were then scanned using GenePix 4000B scanner (Molecular Devices) and the probe intensities were extracted and processed using the GenePix Pro 6.0 software (Molecular Devices). After removing experimental control probes, the intensities of 30968 probes were quantile normalized to have similar distributions across all 4 arrays. The normalized intensity of probes derived from the same gene was collapsed into a single NCBI Entrez gene by the maximal value in each sample (mapping information is obtained from Phalanx's probe annotation table). In total, 15598 genes were measured at transcription level. Differential expression of genes was quantified using fold enrichment. Gene functional annotation regarding cell proliferation, cell apoptosis, CREB signaling, breast cancer signature was downloaded from MSigDB database [38]. Gene set enrichment analysis was described by Mootha [23]. All data is MIAME compliant and the raw data has been deposited in a MIAME complaint database GEO, as detail on the website http://www.ncbi.nlm.nih.gov/geo/query/acc.cgi?token=dngdzomiqwwwwzc&acc=GSE31631. The accession number is GSE31631.

### Sequence of primers for qPCR

*GAPDH* primers: 5′-GAAGGTGAAGGTCGGAGTC-3′ (forward)

5′-GAAGATGGTGATGGGATTTC-3′ (reverse).

*CCND1* primers: 5′-AATGACCCCGCACGATTTC-3′(forward)

5′-TCAGGTTCAGGCCTTGCAC-3′ (reverse)

*BCL2* primers: 5′-ACATCGCCCTGTGGATGACT-3′ (forward)

5′-GGGCCGTACAGTTCCACAAA-3′ (reverse)

*CCNB1* primers: 5′-AGCTGCTGCCTGGTGAAGAG-3′(forward)

5′- GCCATGTTGATCTTCGCCTTA -3′(reverse)

*ROR1* primers: 5′-CAACAAGAAGCCTCCCTAATGG-3′ (forward)

5′-CCTGAGTGACGGCACCTAGAA-3′ (reverse)

*ACTB* primers: 5′-CGAGAAGATGACCCAGATCATGTT-3′ (forward)

5′-CCTCGTAGATGGGCACAGTGT-3′ (reverse)

### The shRNA target sequences of human ROR1

5′-ATCCGGATTGGAATTCCCATG-3′ (ROR1-shRNA1)

5′-CTTTACTAGGAGACGCCAATA-3′ (ROR1-shRNA2, Open Biosystem).

The non-target shRNA or siRNA sequence: 5′-AGCGGACTAAGTCCATTGC-3′

### Small interfering RNA (siRNA) for human CREB1 and CK1ε

5′-GGUGGAAAAUGGACUGGCUtt-3′ (CREB-siRNA, Ambion siRNA, ID# 109994)

5′-CCAGUGUUUGCUUAGUGUCUUCtt-3′ (CK1ε-siRNA)

## Supporting Information

Figure S1 **ROR1 expression is up-regulated in patients with aggressive disease.** (**A–C**) Each dot plot represents median-centered log_2_ expression of ROR1 mRNA by cancer cell lines or tumor tissue from an individual patient. Cancer cell lines or patient samples with similar molecular and phenotypic characteristics are clustered together, as indicated at the bottom of each cluster. Below this designation is indicated the number of distinct cases in each cluster. The line indicates the median ROR1 expression level by the group. P indicates the statistical significance of the differences in the collective ROR1 expression between the two groups, as calculated using Student's t test. The each dataset used is indicated on the top of each graph.
(TIF)

Figure S2 **Silencing ROR1 protein reduces the rate of cell growth.** (**A**) MDA-MB-231 tumor cells were transduced with vectors encoding Ct-shRNA or ROR1-shRNA and then selected for stable expression of the shRNA. Lysates of each cell type as indicated on the bottom selected for stable expression of Ct-shRNA or ROR1-shRNA were examined for ROR1 or β-Actin via immunoblot analyses. The vector used to transduce the cells is indicated at the top of each lane. (**B**) Serum starved MDA-MB-231 cells were labeled with BrdU for 2 hours, and then stained with anti-BrdU antibody and 7-amino-actinomycin. Contour plots of MDA-MB-231 cells (Ct-shRNA, left) or cells silenced for ROR1 (ROR1-shRNA, right) depict the different proportions of cells found within the gated area, which represents cells in S-phase. The height of each bar in the graph on the right indicates the mean proportion of cells in S phase that were BrdU positive in MDA-MB-231 cells transduced with Ct-shRNA or ROR1-shRNA. The error bars indicate the standard error of triplicate samples. P indicates the statistical significance as measured by Student's t test.
(TIF)

Figure S3 **Changes of gene expression profiles of MDA-MB-231 cells following silencing of ROR1.** Expression of 1435 CREB-bound genes (**A**), 193 cell proliferation related genes of CREB-bound genes (**B**), 104 cell apoptosis related genes of CREB-bound genes (**C**), 10 known breast cancer signature genes of CREB-bound genes (**D**) in MDA-MB-231 cells following silencing of ROR1. Numbers under color bars denote log2 fold changes of a gene relative to its mean expression across the 4 arrays. Genes were sorted in a descending order of the average fold-change of control cells over ROR1-shRNA cells. in MDA-MB-231 cells (Ct-shRNA_rep1 and _rep2) and cells silenced for ROR1 (ROR1-shRNA_rep3 and _rep4) are shown. Red indicates genes that are expressed at higher levels by MDA-MB-231 cells relative to MDA-MB-231 cells silenced for expression of ROR1, where green indicates genes with higher expression in cells silenced for ROR1 relative to that of wild-type cells. Black indicates the genes that are expressed at equal levels in ROR1 positive versus ROR1-silenced cells. Numbers under color bars denote log2 fold changes of a gene relative to its mean expression across the 4 arrays. Genes were sorted in a descending order of the average fold-change of control cells over ROR1-shRNA cells. CREB-bound genes and gene functions related to cell proliferation and apoptosis were downloaded from the database of CREB binding on the promoter [38], MSigDB database (Molecular Signature Database) and the database of a gene–expression signature in breast cancer (VandeVijver et al., 2002). We subsequently validated the expression of genes marked by an asterisk (*) via quantitative RT-PCR and immunoblot analyses for the encoded proteins.
(TIF)

Figure S4 **Either silencing CREB or inhibiting CK1ε activity is able to abolish ROR1-induced cell growth.** (**A**) Equal numbers of MDA-MB-231 cells (Ct-shRNA) or ROR1-silenced cells (ROR1-shRNA) were seeded, transfected with control siRNA (Ct-siRNA) or CREB siRNA (CREB-siRNA) and monitored for growth over 72 hours using the WST-8 assay. The mean number of viable cells ± S.E.M. in each condition was provided in the graph. P indicates the statistical significance as assessed by Student's t test. (**B**) Immunoblot analysis of proteins as indicated on the right margin for lysates of MDA-MB-231 cells treated with (+) or without (−) 0.25 µg/ml IC261 for 16 hours. (**C**) MDA-MB-231 cells (diamonds) or ROR1-silenced cells (squares) were treated with increasing doses of IC261, as indicated on the x-axis, and then incubated with WST-8 to assess cell growth at 48 hours. The graphs provide the mean proportion of viable cells, ± S.E.M of triplicate samples. The asterisk indicate concentrations of LY294002 at which significant differences were observed between the two cell populations ((*P<0.05 and **P<0.01).
(TIF)

Figure S5 **Wnt5a protein is expressed in various cancer cell lines and could enhance the tumor cell growth.** (**A**) Expression of Wnt5a in various tumor cell-lines. Total cell lysates of various tumor cell-lines, as indicated at the top of the figure, were examined via immunoblot analyses using antibodies specific for Wnt5a or β-Actin, as indicated on the right margin. (**B**) Equal numbers of MDA-MB-231 cells were plated in replicate wells of 96-well plates and cultured in media containing vehicle or increasing concentrations of recombinant Wnt5a protein (rWnt5a) for 24 hours, as indicated in the legend. The heights of the bars indicate the mean number of cells harvested from triplicate wells at 0 or 24 hours using the WST-8 assay. *P<0.05 by Dunnett's multiple comparisons test.
(TIF)

Figure S6 **Immunoblot analyses for Met or ROR1 expression in lysates from various cancer cells as indicated on the top. β-actin serves as loading control.**
(TIF)

Table S1 **Gene expression of subnetworks involved in CREB regulation in ROR1+ versus ROR1-silenced MDA-MB-231.**
(DOCX)

Table S2 **Gene expression of 70-signature genes in ROR1+ versus ROR1-silenced MDA-MB-231.**
(DOCX)

Table S3 **The correlation of ROR1 expression with p-CREB in breast cancer patients (χ^2^ test, P<0.05).**
(DOCX)
