# Supplementary figures and images for: ROR1 Is Expressed in Human Breast Cancer and Associated with Enhanced Tumor-Cell Growth
Source: PLoS One. 2012 Mar 5;7(3):e31127. doi: 10.1371/journal.pone.0031127 (PMC3293865; doi:10.1371/journal.pone.0031127)

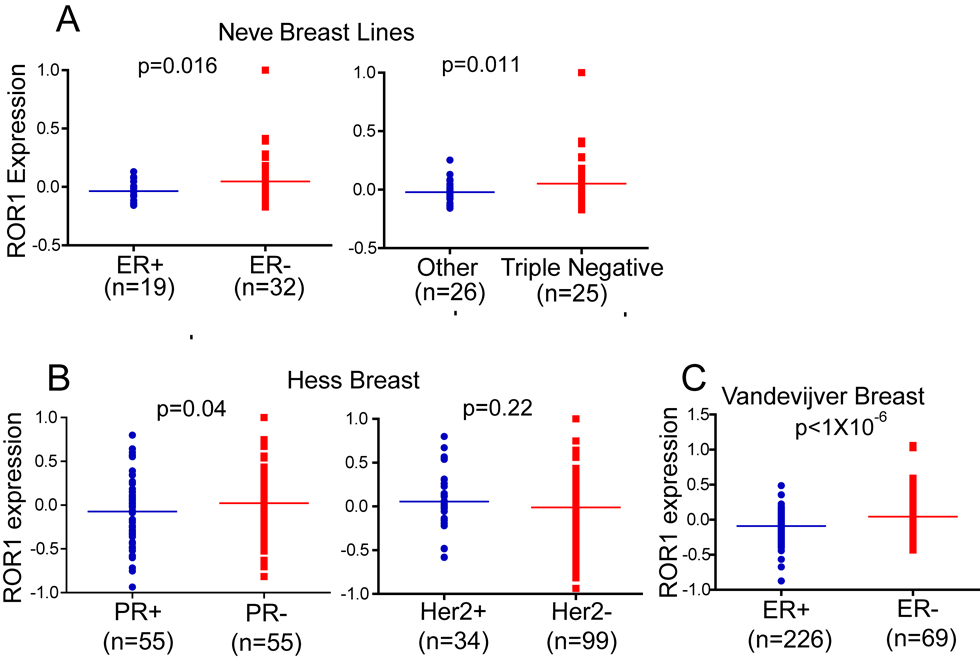

Supplement: Figure S1 — ROR1 expression is up-regulated in patients with aggressive disease. (A–C) Each dot plot represents median-centered log2 expression of ROR1 mRNA by cancer cell lines or tumor tissue from an individual patient. Cancer cell lines or patient samples with similar molecular and phenotypic characteristics are clustered together, as indicated at the bottom of each cluster. Below this designation is indicated the number of distinct cases in each cluster. The line indicates the median ROR1 expression level by the group. P indicates the statistical significance of the differences in the collective ROR1 expression between the two groups, as calculated using Student's t test. The each dataset used is indicated on the top of each graph. (TIF) [file pone.0031127.s001.tif]

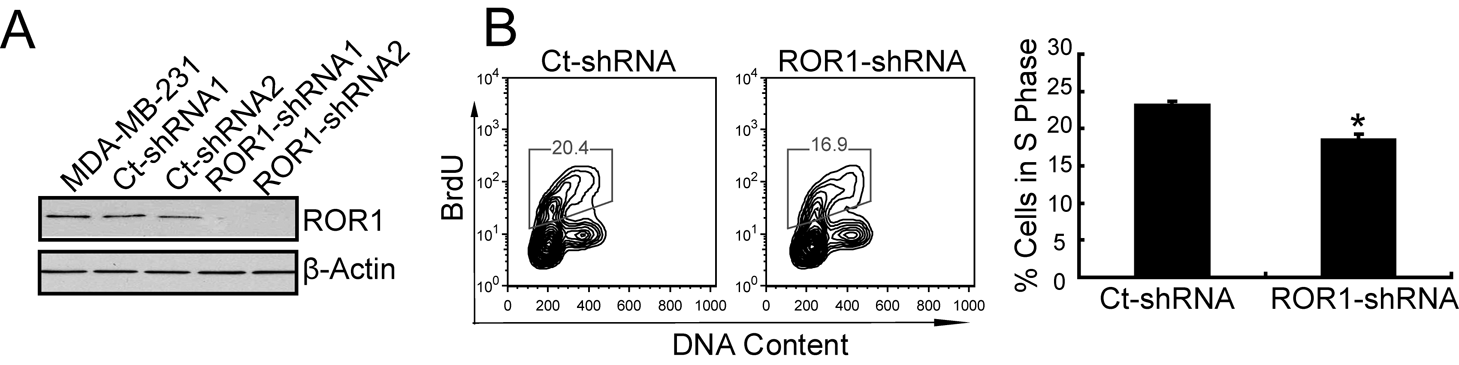

Supplement: Figure S2 — Silencing ROR1 protein reduces the rate of cell growth. (A) MDA-MB-231 tumor cells were transduced with vectors encoding Ct-shRNA or ROR1-shRNA and then selected for stable expression of the shRNA. Lysates of each cell type as indicated on the bottom selected for stable expression of Ct-shRNA or ROR1-shRNA were examined for ROR1 or β-Actin via immunoblot analyses. The vector used to transduce the cells is indicated at the top of each lane. (B) Serum starved MDA-MB-231 cells were labeled with BrdU for 2 hours, and then stained with anti-BrdU antibody and 7-amino-actinomycin. Contour plots of MDA-MB-231 cells (Ct-shRNA, left) or cells silenced for ROR1 (ROR1-shRNA, right) depict the different proportions of cells found within the gated area, which represents cells in S-phase. The height of each bar in the graph on the right indicates the mean proportion of cells in S phase that were BrdU positive in MDA-MB-231 cells transduced with Ct-shRNA or ROR1-shRNA. The error bars indicate the standard error of triplicate samples. P indicates the statistical significance as measured by Student's t test. (TIF) [file pone.0031127.s002.tif]

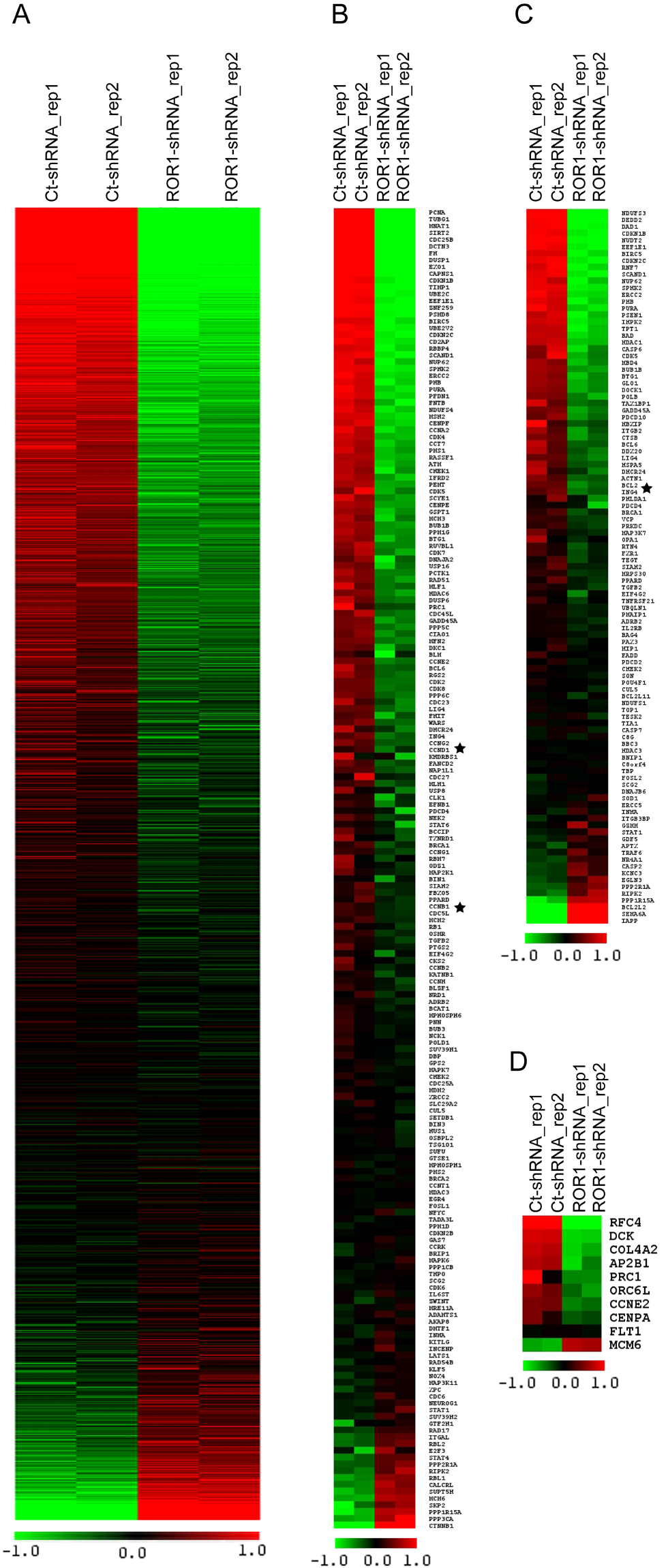

Supplement: Figure S3 — Changes of gene expression profiles of MDA-MB-231 cells following silencing of ROR1. Expression of 1435 CREB-bound genes (A), 193 cell proliferation related genes of CREB-bound genes (B), 104 cell apoptosis related genes of CREB-bound genes (C), 10 known breast cancer signature genes of CREB-bound genes (D) in MDA-MB-231 cells following silencing of ROR1. Numbers under color bars denote log2 fold changes of a gene relative to its mean expression across the 4 arrays. Genes were sorted in a descending order of the average fold-change of control cells over ROR1-shRNA cells. in MDA-MB-231 cells (Ct-shRNA_rep1 and _rep2) and cells silenced for ROR1 (ROR1-shRNA_rep3 and _rep4) are shown. Red indicates genes that are expressed at higher levels by MDA-MB-231 cells relative to MDA-MB-231 cells silenced for expression of ROR1, where green indicates genes with higher expression in cells silenced for ROR1 relative to that of wild-type cells. Black indicates the genes that are expressed at equal levels in ROR1 positive versus ROR1-silenced cells. Numbers under color bars denote log2 fold changes of a gene relative to its mean expression across the 4 arrays. Genes were sorted in a descending order of the average fold-change of control cells over ROR1-shRNA cells. CREB-bound genes and gene functions related to cell proliferation and apoptosis were downloaded from the database of CREB binding on the promoter [38], MSigDB database (Molecular Signature Database) and the database of a gene–expression signature in breast cancer (VandeVijver et al., 2002). We subsequently validated the expression of genes marked by an asterisk (*) via quantitative RT-PCR and immunoblot analyses for the encoded proteins. (TIF) [file pone.0031127.s003.tif]

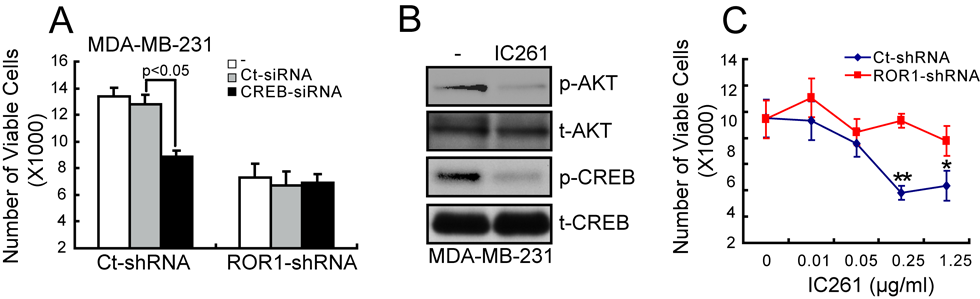

Supplement: Figure S4 — Either silencing CREB or inhibiting CK1ε activity is able to abolish ROR1-induced cell growth. (A) Equal numbers of MDA-MB-231 cells (Ct-shRNA) or ROR1-silenced cells (ROR1-shRNA) were seeded, transfected with control siRNA (Ct-siRNA) or CREB siRNA (CREB-siRNA) and monitored for growth over 72 hours using the WST-8 assay. The mean number of viable cells ± S.E.M. in each condition was provided in the graph. P indicates the statistical significance as assessed by Student's t test. (B) Immunoblot analysis of proteins as indicated on the right margin for lysates of MDA-MB-231 cells treated with (+) or without (−) 0.25 µg/ml IC261 for 16 hours. (C) MDA-MB-231 cells (diamonds) or ROR1-silenced cells (squares) were treated with increasing doses of IC261, as indicated on the x-axis, and then incubated with WST-8 to assess cell growth at 48 hours. The graphs provide the mean proportion of viable cells, ± S.E.M of triplicate samples. The asterisk indicate concentrations of LY294002 at which significant differences were observed between the two cell populations ((*P<0.05 and **P<0.01). (TIF) [file pone.0031127.s004.tif]

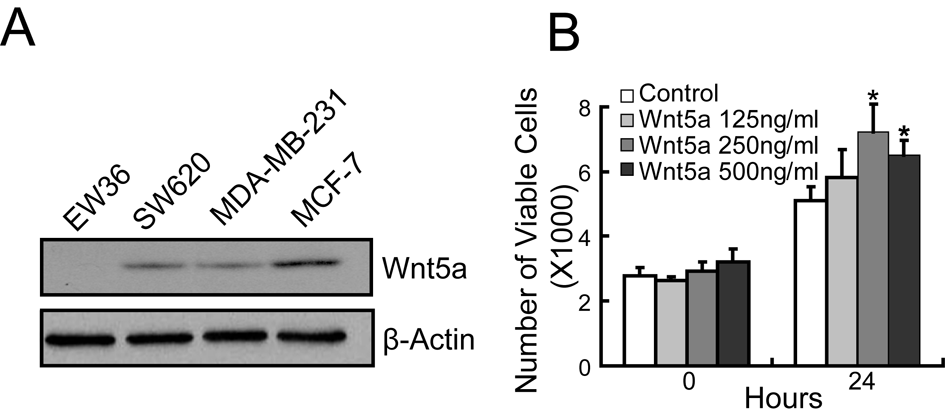

Supplement: Figure S5 — Wnt5a protein is expressed in various cancer cell lines and could enhance the tumor cell growth. (A) Expression of Wnt5a in various tumor cell-lines. Total cell lysates of various tumor cell-lines, as indicated at the top of the figure, were examined via immunoblot analyses using antibodies specific for Wnt5a or β-Actin, as indicated on the right margin. (B) Equal numbers of MDA-MB-231 cells were plated in replicate wells of 96-well plates and cultured in media containing vehicle or increasing concentrations of recombinant Wnt5a protein (rWnt5a) for 24 hours, as indicated in the legend. The heights of the bars indicate the mean number of cells harvested from triplicate wells at 0 or 24 hours using the WST-8 assay. *P<0.05 by Dunnett's multiple comparisons test. (TIF) [file pone.0031127.s005.tif]

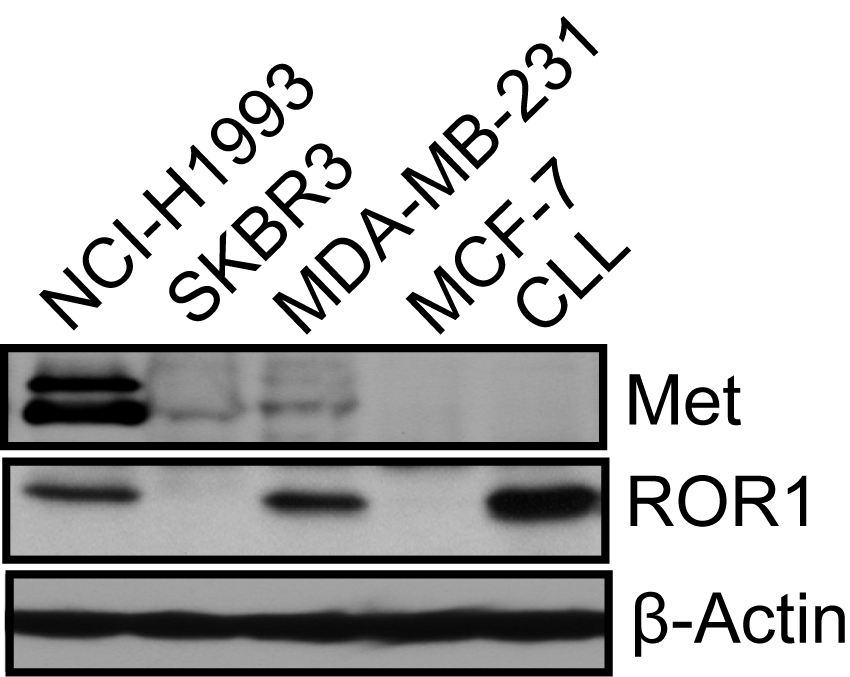

Supplement: Figure S6 — Immunoblot analyses for Met or ROR1 expression in lysates from various cancer cells as indicated on the top. β-actin serves as loading control. (TIF) [file pone.0031127.s006.tif]
