# Supplementary material for: ROR1 Is Expressed in Human Breast Cancer and Associated with Enhanced Tumor-Cell Growth
Source: PLoS One. 2012 Mar 5;7(3):e31127. doi: 10.1371/journal.pone.0031127 (PMC3293865; doi:10.1371/journal.pone.0031127)
Supplement: Table S1 — Gene expression of subnetworks involved in CREB regulation in ROR1+ versus ROR1-silenced MDA-MB-231. (DOCX) [file pone.0031127.s007.docx]

**Table S1. Gene expression of subnetworks involved in CREB regulation in ROR1+ versus ROR1-silenced MDA-MB-231**

| Gene Subnetworks | Number Of Genes Found On Array | Number Of Genes With ≥1.5-fold Increase in ROR1 Positive Cells Relative To ROR1-negative Cells  (Frequency) | Number Of Genes With ≥1.5-fold Decrease In ROR1 Positive Cells Relative To ROR1-negative Cells  (Frequency) |
| --- | --- | --- | --- |
| CREB-bound genes | 1435 | 549 (0.38) | 135 (0.09) |
| CREB-bound genes related to proliferation | 193 | 87 (0.45) | 14 (0.07) |
| CREB-bound genes related to apoptosis | 104 | 42 (0.40) | 9 (0.09) |
