# Supplementary material for: ROR1 Is Expressed in Human Breast Cancer and Associated with Enhanced Tumor-Cell Growth
Source: PLoS One. 2012 Mar 5;7(3):e31127. doi: 10.1371/journal.pone.0031127 (PMC3293865; doi:10.1371/journal.pone.0031127)
Supplement: Table S2 — Gene expression of 70-signature genes in ROR1+ versus ROR1-silenced MDA-MB-231. (DOCX) [file pone.0031127.s008.docx]

**Table S2. Gene expression of 70-signature genes in ROR1+ versus ROR1-silenced MDA-MB-231**

| Gene Subnetworks | Number of known Genes | Number Of known Genes Found On Array | Number of CREB-bound genes  (Frequency) | Number Of CREB-bound Genes With ≥1.5-fold Increase in ROR1 Positive Cells Relative To ROR1-negative Cells  (Frequency) | Number Of CREB-Genes With ≥1.5-fold Decrease In ROR1 Positive Cells Relative To ROR1-negative Cells  (Frequency) |
| --- | --- | --- | --- | --- | --- |
| 70-signature genes | 49 | 29 | 10 (0.34) | 7 (0.70) | 1(0.10) |
