# Supplementary material for: ROR1 Is Expressed in Human Breast Cancer and Associated with Enhanced Tumor-Cell Growth
Source: PLoS One. 2012 Mar 5;7(3):e31127. doi: 10.1371/journal.pone.0031127 (PMC3293865; doi:10.1371/journal.pone.0031127)
Supplement: Table S3 — The correlation of ROR1 expression with p-CREB in breast cancer patients (χ2 test, P<0.05). (DOCX) [file pone.0031127.s009.docx]

**Table S3. The correlation of ROR1 expression with p-CREB in breast cancer patients. (χ^2^ test, P <0.05)**

| p-CREB  ROR1 | Score 0 | Score 1 | Score 2 |
| --- | --- | --- | --- |
| Score 0 | 6 | 1 | 0 |
| Score 1 | 5 | 7 | 0 |
| Score 2 | 5 | 8 | 4 |
